# Supplementary material for: Patterns of Intron Gain and Loss in Fungi
Source: PLoS Biol. 2004 Nov 30;2(12):e422. doi: 10.1371/journal.pbio.0020422 (PMC532390; doi:10.1371/journal.pbio.0020422)
Supplement: Table S1 — Also available at http://genes.mit.edu/NielsenEtAl/. (4.3 MB ZIP). [file pbio.0020422.st001.zip › NielsenEtAl/html/205.html]

AN0077.1.NCU01364.1.MG08311.1.FG07012.1


```
 CLUSTAL W (1.82) Multiple Sequence Alignments - Introns Inserted


Sequence 1: NCU01364.1	359 aa
Sequence 2: MG08311.1	352 aa
Sequence 3: FG07012.1	364 aa
Sequence 4: AN0077.1	318 aa
Alignment Length: 401 aa
Number Identitical Residues: 120 aa
Alignment Score (without introns) 6476


MG08311.1 	------------------MATAAKIPVIDLAS--KDEAQVAKDLVDAAVEHGFVYIKNQG
NCU01364.1	------------------MTGVAKIPVIDISNDNQDQARVAKDLVEAAIEHGFIYIKNTG
FG07012.1 	MNFPMKDKLGHFKRPLGIDQRTANIPLIDISAPDADQEEIARRLVDAAEEHGFIYIRNLG
AN0077.1  	------------MAEPTPAGTSIELPILDISN--PEDPAVGEAMLDAATKYGFLYVDSKG
          	                       ::*::*::    ::  :.. :::** ::**:*: . *

MG08311.1 	NDIPVDAIDGAFDL0ARKLFAS-PMEEKASCPIQKD~NRG~YSGMHVETLDPANQR~VGD
NCU01364.1	KDIPVEDVDAAFEL0ARKLFKETPVEEKQACTIQKN~NRG0WSAMHYETLDPKNQR0VGD
FG07012.1 	HDIQAKDVDGAFDL0VRKTFEC-SVDEKQRCTIQTN~NRG~WSGMHSETLDPKNQK0IGD
AN0077.1  	TDFTAEDVRKAFE-~--------------------R0NRG~WSGMHSETLDPEHQR0TGD
          	 *: .. :  **:                        *** :*.** ***** :*:  **

MG08311.1 	FKE2AFNFGECKD--GKPQQPLPPALTPHAHQIGAFFDYCYRLALRLNTLLGIGLA~TDP
NCU01364.1	FKE2AFNFGEPSQQGSHLQQPIPPTIRSAEPFISRFHTLCHHLSLRLNTLLGQGLS~VSP
FG07012.1 	FKE2AFNFGEFTD--GKAQQPLPSDLVPDEPQISAFADSCHDLCQKLLYLLGLGLG0VG-
AN0077.1  	FKE2AFNFGEFTA-EGKAQQPLPPALAPHEAEIADFASLCTKTCNRILSLLSLGLK0TAD
          	*** ****** .   .: ***:*. : .    *. *   *   . ::  **. **  .  

MG08311.1 	PSFFRDAHLRERGPSGSILRMLHYPPLGDAAGAS-----ADDVRAGAHSDYGSLTLLFRL
NCU01364.1	PDFFTSAHLRENGASGTILRLLYYPPAPSSLTAEDKKGDGDDVRAGAHSDYGSMTLLFRL
FG07012.1 	-DFFSSAHNTDKGASSSILRFLRYPPPESTAHTE------EDVRAGSHSDYGSITLLFRL
AN0077.1  	YDHTV------------------------------------DVRAGAHSDYGSITLLFQR
          	 ..                                      *****:******:****: 

MG08311.1 	RGQAGLEILTPEGSWAPVPVTP---PGTEDDPS-----PPILVNIGDLLSYWTAGLLRST
NCU01364.1	KGQAGLEIQTREGNWVPVPVCP---PGTETDPA-----PPILVNIGDLLSYWTNGLLRST
FG07012.1 	KGQAGLEVLTKDNVWAPVPVCP---AGTEDDPS-----PPILINIGDLLSYWTNGLFRST
AN0077.1  	PGQPGLEILTPEGTWAPVAVEPGRPSPSGDDVNNEFAFPPILVNIGDLLSYWTDGLLKST
          	 **.***: * :. *.**.* *. .. :  *  .. : ****:********** **::**

MG08311.1 	VHRVVFPVAGG---AEGETAVDPRYSMAFFCHPVGNAALEPVPSERVRAEGLK-------
NCU01364.1	VHRVVFPGAAGSSTVEGETGGEERYSIAYFCHPVGTMKLEPVPSERVRAFGEE-------
FG07012.1 	LHRVVFPTEGSVR-VQGETNEGPRYSIAYFCHPVGTVPLEPVPSERVKNFVPA-------
AN0077.1  	VHRVVFPASER----TGNSSQD-RYSIVFFCHPVDSTELVPVPSDVVKAYREEKVVDAEE
          	:******         *::    ***:.:*****..  * ****: *:     .  .:..

MG08311.1 	---AAAREGNPHALKKDEILTADEHLQMR~LRASYAGLYGDEGKKRCQ
NCU01364.1	---QNGK-GVLNGERK--MLTADEHLNMR2LQASYLKLYEDEKKE---
FG07012.1 	---EGAPSENPYATSK--VMTADEHLFMR~LKESYGDLYDKKS-----
AN0077.1  	RVGFGGGAGNLEPGRR--TLTAAEHLAMR~LNATYDFRQEATVA----
          	  .  .         :   :** *** ** *. :*
```
